# A real-world information security performance assessment using a multidimensional socio-technical approach

**DOI:** 10.1371/journal.pone.0238739

**Published:** 2020-09-08

**Authors:** Kaja Prislan, Anže Mihelič, Igor Bernik

**Affiliations:** Faculty of Criminal Justice and Security, University of Maribor, Ljubljana, Slovenia; Xidian University, CHINA

## Abstract

Measuring the performance of information security is an essential part of the information security management system within organisations. Studies in the past mainly focused on establishing qualitative measurement approaches. Since these can lead to ambiguous conclusions, quantitative metrics are being increasingly proposed as a useful alternative. Nevertheless, the literature on quantitative approaches remains scarce. Thus, studies on the evaluation of information security performance are challenging, especially since many approaches are not tested in organisational settings. The paper aims to validate the model used for evaluating the performance of information security management system through a multidimensional socio-technical approach, in a real-world settings among medium-sized enterprises in Slovenia. The results indicate that information security is strategically defined and compliant, however, measures are primarily implemented at technical and operational levels, while its strategic management remains underdeveloped. We found that the biggest issues are related to information resources and risk management, where information security measurement-related activities proved to be particularly problematic. Even though enterprises do possess certain information security capabilities and are aware of the importance of information security, their current practices make it difficult for them to keep up with the fast-paced technological and security trends.

## 1 Introduction

An effective information security management system (ISMS) can minimise business risks, maximise return on investments, facilitate business opportunities, support legal compliance, and boost commercial image and competitive edge of organisations [1, 2]. To ensure a successful protection of information assets and a stable information security management (ISM), organisations must perform a security assessment, analyse their information system processes and develop ISM accordingly [3–6]. Thus, the performance of the ISMS must be continuously monitored and analysed. If necessary, appropriate preventive and/or corrective actions should be taken [1].

Although information security (ISec) performance measurement is recognised as an important element of the ISMS, many challenges and issues persist and hinder the development of ISec in organisations. The current state-of-play in ISec metrics does not seem sufficiently advanced in practice since it remains mostly ad-hoc due to its inherent complexity [7, 8]. This leaves management decisions to be based on heuristics and optimistic perceptions [5]. Studies show that only few organisations develop metrics and systematically and continuously measure their posture, performance and, progress in the area of information security [9–13]. Most organisations do not have adequate tools or training to verify whether their organisation's practices are compliant with recommended guidelines [14]. Furthermore, organisations are also faced with a dilemma of how to ensure comprehensive measurement throughout the organisation and prove the effectiveness of the entire ISMS [15]. Since information security is a complex and multidimensional system with an enormous scope and volume of relevant data, ISec professionals are often overwhelmed and unable to develop effective assessment processes [16]. Hence, security managers mostly focus on technical goals and controls, while only few are capable of performing comprehensive multidimensional ISMS assessments down to the last level [15, 17].

Fifty percent of all attacks on organisations are directed at smaller enterprises and are becoming a frequent target of cyber criminals [18]. Despite the higher number of incidents at the level of large organisations, the ISec capabilities of smaller organisations are often less developed, which is why small- and medium-sized enterprises (SMEs) have been recording the strongest increase in costs and other consequences generated by ISec incidents [9, 12]. For instance, almost half of SMEs still believe that information security is an (unnecessary) expense, which hinders the adequate development of capabilities in this field [19]. Thus, smaller organisations have greater problems when monitoring the effectiveness of information security. They usually use less formal and sophisticated procedures and have fewer high-quality information at their disposal, which is why they have more difficulties in anticipating and predicting security incidents [20, 21]. Therefore, it is necessary to focus current endeavours and support on smaller organisations, where the situation seems most critical and is consequently affecting the general security situation in a business environment through inter-organisational and partner relations. On the other hand, it is much easier to measure and evaluate their systems by applying predefined models and criteria, since these tend to be less complex and require less intricate management processes [22].

In recent years, noticeable theoretical progress has been made in advancing the areas of ISec measurement and reporting, however, several gaps remain [23]. Studies in the past mainly focused on establishing qualitative measurement approaches. To avoid qualitative metrics leading to ambiguous conclusions, quantitative metrics are being increasingly proposed as a useful alternative. Nevertheless, the literature on quantitative approaches is scarce leaving this kind of studies challenging [8, 24, 25]. Literature review suggests that evidence-driven metrologies based on comprehensive frameworks that would enable informed and effective decision making are needed [6, 7, 26]. To address this issue, we conducted a research among medium-sized enterprises. The participating units were selected based on a systematic sampling through a nation-wide database of business enterprises. The research enabled us to validate a model for evaluating the performance of information security management system through a multidimensional socio-technical approach (i.e. ISP 10×10M) in real-world settings.

The paper is structured as follows. In the next subsections, we discuss related work and motivation for our research. The characteristics and factors comprising the ISP 10×10M model are presented in the Section 2. In the Section 3, we present the methodology of the research and the results in the Section 4. The results are discussed in the Section 5, while the Section 6 draws conclusions.

### 1.1 Related work

Two types of quantitative approaches related to information security performance are being developed that differ in terms of their measurement focus and orientation. Firstly, assessments that evaluate the ISMS performance in terms of ISec risks are based on threats and technical vulnerabilities, however, it is difficult to ensure the sustainability of such approaches due to the changing nature of threat landscape and constant technological advancements [5]. In such circumstances, dynamic threat values and manoeuvrable frameworks are necessary. Secondly, control-centric metrics based on established standards are proposed as an alternative. Such an approach, which enables assessments against various requirements, has been recognised as an efficient, flexible, and sustainable mean of measuring ISec effectiveness [26]. Herein, the effectiveness of ISMS is evaluated in terms of the level of security measures implementation strength (i.e. development level), which in turn enables a control-gap analysis and produces quantifiable and operable results [24, 27, 28].

Since our study focuses on the second type of approaches described above, frameworks that provide an assessment of the information security management capabilities are reviewed in the following [29] presented a framework for the assessment of Information Security Governance and Management (ISGM) maturity, consisting of six building blocks (technical security, resource management, risk control, data administration, business continuity management). The framework is a component of a broader IT-CMF (IT Capability Maturity) framework and enables organisations to self-assess the maturity of each building-block and calculate average maturity score. The paper builds on a framework already developed by Innovation Value Institute and provides a hypothetical example of the model use.

A capability assessment framework (CAFISGO) for the information security governance in organizations was developed by [30]. It consists of five key areas (information security governance strategy and metrics; technical asset security management; information service/system/data security management; vulnerability and risk management; information security governance control/compliance/continuity management), 21 security objectives and 80 weighted controls each having a designated weighting coefficient. The method was applied to a large organization in Morocco by using an online survey. The findings indicate that organisation included in their in case analysis is less mature in the areas of security budgeting, resource effectiveness, security threat profiling, and security risk handling [30].

Similarly, [31] found the biggest gap in the organisation's contingency plan/disaster recovery plan and vulnerability management. Using the activities included in the OCTAVE Catalog of practices [31] demonstrated an approach to the evaluation of information risk management maturity. The method focuses on the evaluation of 17 risk management related areas (such as security awareness and training, strategy, management, policies, physical security, etc) and enables to determine maturity for each area as well as overall maturity score. The proposed approach was validated with an exploratory case study including interviews, observations, and document reviews in an IT Department at an industrial automation company in Indonesia.

A tool to assess information security compliance with 27002 information security controls was presented by [32]. The method is based on self-assessment where the organisation selects which controls have already been implemented (yes/no options), however, the method also requires an input of all organisation information assets, which is a time-consuming task. The proposed system enables to determine the critical risks, compliance status and missing countermeasures. The system was evaluated with a middle-sized organization in Austria specialized for secure IT solutions.

A quantitative methodology for the Information Security Maturity Level (ISML) determination was proposed by [33]. The methodology requires a survey, social engineering experiment, and vulnerability assessment implementation for the analysis of three functional areas (people, processes, and technology). The method was tested in four city councils, using two out of three methods they proposed; a survey and a phishing campaign. They found that user, policy, and procedures management are a problem within organisations covered in their research [33].

To develop an instrument (SECURQUAL) that enables an assessment of the effectiveness of enterprise information security programs, the COBIT framework was used [34]. The method relates the information security effectiveness with the number of identified noncompliance issues, a number of security-related internal control weaknesses, and a number of incidents, and 18 ISM processes (people, configuration & change management, monitoring, governance, availability- focused processes). The feasibility of the proposed method was used in a survey among 111 internal and external auditors who evaluated the information security effectiveness of organisations, however, organisations themselves were not a part of the validation. The results of the study showed that non-compliance and internal control weaknesses negatively correlate with information security performance, while previous experience with incidents has a positive effect [34].

In addition to the general models we have described, other ISec measurement models [7, 8, 25, 26, 28, 35, 36] mainly focus either on performance in specific dimensions (e.g. security strategy, security policy, employee compliance; vulnerability management; software or cloud systems security) or on the performance of particular industries/business sectors (e.g. power industry, health-care organisations, state agencies). For example, an approach to measuring organisations' cybersecurity maturity was proposed by [37]. The assessment is conducted with a self-reported questionnaire referring to six areas (Management, Frameworks and Standards, Network, Infrastructure, Awareness and User Management, and Application Security) each consisting of four to five controls. The proposed framework is devised for utility companies only and was not validated in organisational settings. The information security maturity model for critical infrastructure based on CMMI framework for software engineering was proposed by [38]. The method is based on self-evaluation, while the model consists of five maturity levels, five dimensions, and 185 controls deriving from ISO 27001 and 27002, NIST 800–53, and ISA 62443. Their method was evaluated by practitioners from five thermal power plants in Korea. Similarly, a method for Information security management system assessment was developed for the needs of public administration by [39]. They defined five factors important for the information security management process (legal, procedural and organisational, physical and technical, social and resources/capital). A survey questionnaire was then used in 50 public administration units in Poland. The results showed that the biggest issues were related to the lack of information security policy, limited use of risk management, and inadequate vulnerabilities and information security incidents management.

### 1.2 Motivation

A review of the quantitative models shows that they are based on similar methods. Information security management assessment is usually performed through self-reported surveys, in which organizations assess their compliance with specific measures and activities. However, several do not provide an overall assessment score and lack of information about the practical use of the proposed methodology. The majority of identified approaches that provide the guidelines for using their model are not based on weighting the importance of included measures. Hence, they are not sensitive to the level of the importance that different information security management activities may have for overall performance.

A great part of existing models is narrowly focused, either on the assessment of specific ISec areas or enable risk assessment only. Many proposed frameworks are also industry-tailored and deriving from sector-specific guidelines, while comprehensive and uniform models remain underdeveloped.

Moreover, the literature reviewindicates that the practical validation of proposed methods in real-world organisational settings is rare. Several proposed models are theoretical and were not validated, while others mainly base their validation on single case studies, specific industry settings, or professional opinion. Thus, only a few models were able to build on their theoretical value. As a result, empirical assessments and validations of such proposed models are scarce, leaving practical implications dubious. To the best of our knowledge, there was no research performed in a broader network of organisations, with an assessment method that would cover all the above criteria and enable benchmarking and comparison of information security performance between entities.

The current paper builds on our previous work. The aim is to validate the model used for evaluating the quality of ISMS, i.e. the Information Security Performance 10 by 10 Model (ISP 10×10M), that we proposed in [40]. The model may be used for internal evaluations aimed at establishing the key gaps of existing approaches adopted by organisations. The application of the model in different organisational settings also allows for a multiple-case analysis for different purposes, such as benchmarking and the identification of front-runner characteristics. We conducted a research, in which the proposed model was used on a sample of enterprises to evaluate the (then) current state-of-play and identify shortcomings related to the ISM. Based on results obtained in the scope of the study, the paper will provide answers to the following research questions:

**RQ1:** Which information security areas are most/least developed in medium-sized enterprises?
**RQ2:** Which are the information security areas that distinguish enterprises considered as front-runners from other enterprises?

## 2 The ISP 10×10M model

It is commonly established that well-designed quantitative ISec metrics should be subject to rigorous scientific empirical validation, and reflect and/or include the following characteristics:

- a multidimensional security index measurement system (i.e. an approach providing security-related evidence from different viewpoints) enabling the quantification of the results [8, 28];
- top-management enablement to categorise the current ISec level of the organisation into pre-defined performance levels, thus the target performance level can be determined, and monitoring of the progress performed [25];
- a socio-technical design that can provide a uniform perspective and holistic view on the area, illustrating gaps at different levels [23];
- an assessment and analysis process based on multicriteria analysis [41];
- an objective translation of qualitative data into quantifiable/numeric results [42];
- the prioritisation of different ISec areas, i.e. a scoring scheme that assigns a value to each control and defines a weight index for different ISec domains [43–45];
- the calculation of overall scores based on defined indicators [46].

To achieve significant measurement results and enable constant improvements, relevant key performance indicators (KPI) must be carefully defined. Several researchers proposed their selection of KPIs based on regulatory requirements and established international (information security) standards and frameworks (such as ISO/IEC 27k, NIST, COBIT and other industry-specific family of standards) [3, 4, 43, 44, 46–48].

In addition, ISec measurement must also ensure a proper visualisation and representation of results to facilitate informed decision-making by the management [7, 36]. Managers should be able to obtain information about specific gaps and the overall security posture, i.e. the “degree of security” [7, 25, 28, 36]. Hence, the output must help managers to set targets, select the best corrective measures, prioritise the choices/measures, and justify spending resources [25, 36].

**Fig 1:**
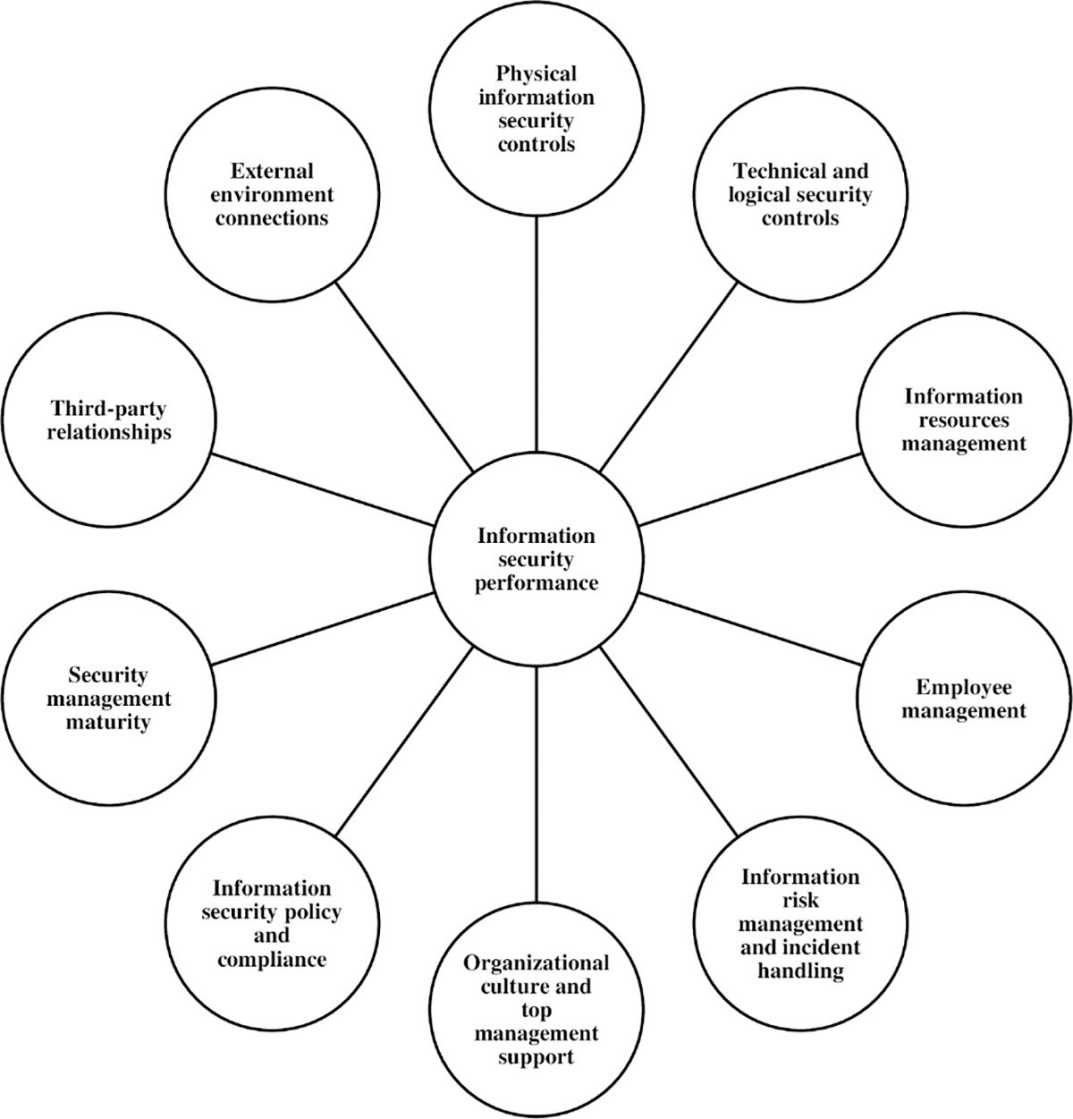
The ISP 10×10M model [40].

The ISP 10×10M model respects the aforementioned recommendations regarding the qualities of measurement approaches. The following subsection presents and discusses ISec areas (factors) included in the model (Fig 1). Their relevance and indispensability for providing a high degree of information security in organisations have been highlighted by internationally accepted standards (namely NIST SP 800–55 & 800–53 [49, 50]; ISO/IEC 27001 and ISO/IEC 27002 [51, 52]; Critical Security Controls (CSC) by the Center for Internet Security [53]; ISACA’s COBIT 5 for Information Security and Business Model for Information Security (BMIS) [54, 55]; PAS 555 by the British Standard Institution (BSI) [56]; The Standard for Information Assurance for Small and Medium-Sized Enterprises by the IASME Consortium [57]), and other relevant literature. The methodology of the model derives from an empirical research conducted among ISec professionals [40]. The model follows the methodology proposed by several other established models such as the Security Effectiveness Score by the Ponemon Institute [58], the Security Operations Maturity Model by Hewlet-Packard [59] and the Capability Maturity Model Integration by the Carnegie Mellon University [60].

### 2.1 The ISP 10×10M areas

The ISP 10×10M model is composed of ten critical success factors (ISec areas), 100 key performance indicators (items) and 3 principal performance levels divided into 6 sublevels (1 –Basic level (1.1 Reactive & Ad-hoc; 1.2 Elementary & Disorganised); 2 –Intermediate level (2.3 Regulated & Compliant; 2.4 Formalised & Standardised); 3 –Advanced level (3.5 Evaluated & Anticipated; 3.6 Proactive & Up-to-date)). The areas composing the model are presented in the following (F1 to F10).

#### F1: Physical information security controls

Physical security measures involve various physical barriers, which are aimed at limiting access to certain environments, buildings or premises, protecting information sources from loss and damage, and disabling access to sources with a view of preventing their abuse or unauthorised use. Physical security measures should be implemented inside (internal physical security controls) as well as outside of the organisations’ buildings (external physical security controls) [61, 62]. External physical security controls include measures aimed at supervising and protecting the areas surrounding a building (e.g. anti-break-in security measures; active and regular control activities at access points, secure location of critical information systems [51, 52, 62]). The key internal physical security controls include the supervision and control of entry points (e.g. access controls, secure storage and maintenance of data, documents, and hardware, protection against accidents and disasters [50–53, 63, 64]).

#### F2: Technical and logical security controls

Technical and logical ISec measures include software-based or technical restrictions aimed at controlling andrestricting activitiesperformed by users when using hardware, data, network, or applications [65]. These measures are used to control information systems, detect anomalies, and prevent unauthorized activities. The key logical controls consist of advanced systems for the detection and mitigation of anomalies and threats, solutions for managing digital identities and user rights, restrictions related to the use of ICT; and the security of network components and online services [51–53, 57, 63–67].

#### F3: Information resources management

Measures aimed at providing the security of information resources are implemented to ensure the confidentiality and integrity of information, at the stage of their creation, storage, processing, transmission, and destruction. To guarantee the security and protection of information, it is important to first provide for the adequate capability and capacity of information systems enabling a smooth and reliable information processing and management [68]. Information resources management controls also include data encryption; data backups; backup locations; isolation of sensitive systems; safe and secure destruction of devices and data; central system management and configuration management; secure remote access; testing of systems and applications in secure environments; patch management; and mobile device management [51, 53, 57, 61, 63, 64, 67].

#### F4: Employee management

The human resources that must be taken into consideration when planning and implementing security measures include all end-users, former and current employees, as well as contractual or temporary staff [69, 70], who may have malicious motives, lack competences for using ICT or are negligent [71]. Primarily, organisations must provide an adequate level of ISec awareness among all users, which means that employees must understand the importance of information security for the organisation, be familiar with the rules governing the protection of information resources, and have the required competences for the proper and safe use of ICT [72, 73]. Motivation and the willingness of employees to participate in education and training processes is greatly dependent on the education and training approaches whichshould be adapted to users’ interests and existing level of knowledge [74]. Furthermore, key employee management controls consist of measures, which are implemented before, during, and after employment or formal cooperation (e.g. integrity checks; formal commitment to compliance; assessing compliant behaviour; user behaviour analytics; need-to-know policy; a fully functioning disciplinary regime [13, 52, 57, 61]).

#### F5: Information risk management and incident handling

The main purpose of the information risk management process is to identify security risks and reduce them to an acceptable level [75, 76]. Here, the objective is to provide effective security, which is rational and cost-efficient, thus striking the right balance between risks on one hand and investments required for the implementation of security and protection measures on the other [64]. This entails a systematic procedure, in the scope of which organisations identify and assess information resources in terms of their value, vulnerability, and threats, decide on the acceptability of risks, and the ways of tackling them [64, 77, 78]. Apart from risk analysis and assessment procedures, key information risk management controls also include business continuity and incident response plans, as well as recovery and normalisation procedures in the event of threat realisation [55, 57, 64].

#### F6: Organisational culture and top management support

The responsibility for information security is primarily in the hands of executive board members and strategic management, which ensure this domain is developing as a business function [79]. Thus, the ISM must receive adequate organisational support both at the strategic as well as at the operational level. The management’s principal task is to provide a sound approach and positive example to all users (top-down approach) [61, 80]. Successful ISM also requires continuous and regular two-sided (vertically and horizontally) communication and the coordination of needs expressed by various stakeholders. All key interest groups should receive regular information about the current state-of-play and issues, while the ISec topics should be regularly discussed with users [81].

#### F7: Information security policy and compliance

The effectiveness of information security depends on a proper approach to the establishment of security objectives. Such an approach is inextricably linked to a well-defined ISec strategy and policy, which support general business goals. The strategy, which lays down fundamental objectives, guidelines, and plans, serves as a basis for drafting and adopting an ISec policy, which prescribes rules and specific goals of the ISec programme [81, 82]. This is the backbone of all management activities and the basis of a sound ISec plan; in fact, it is the main tool used by security management [83]. The policy must define the prohibited actions and consider the needs of both users and business processes in order to ensure the maximum functionality of systems [63, 84]. From a strategical point of view, ISM must also include compliance with legislative and other statutory requirements (for instance contractual and international provisions) [52, 54]. Apart from regulatory, compliance with other binding documents (e.g. legally and formally binding treaties, agreements, and contracts) is also important [52, 57, 61, 62, 85]. In broader terms, compliance entails conformity with established standards, which may not be legally binding, but represent a set of recommendations that should be respected.

#### F8: Security management maturity

Security management must show an adequate level of maturity to be able to provide adequate information security [13, 59, 70, 86, 87]. Security management maturity depends on theorganisational structure (it is recommended that information security is developed as a separate business function [88–91]), as well as on the ability to sustain positive attitudes towards the security policy throughout the organisation. Apart from an adequate system of controls, social preventive measures (e.g. management of informal rules [81], organisational security culture [73], ISec legitimacy [71]) must be developed to address various factors that influence people’s attitudes and behaviours. In this respect, social relationships and experiences gained by employees in their workplace play an important role, as they influence employees’ attitudes towards information security and related rules, which in turn predisposes their willingness to respect and comply with security rules and regulations [92].

#### F9: Third-party relationships

Organisations’ ability to provide information security depends on the security of all related parties. Therefore, organisations must identify weak links not only in their own environment but also in their business relationships. The main issue stems from the fact that the ISec posture in cooperating organisations is often disproportionate, as some organisations have more developed security abilities and awareness than others [21, 70]. Thus, the model of trust should be established together with related parties [12, 70]. Related parties must be supported in improving their security abilities and capabilities, as well as in setting up an ISec system that would be able to protect inter-organisationalsystems. The range of related parties creating the business ecosystem is rather broad, e.g. partner organisations, expert and advisory groups, suppliers, and customers [69]. For the security of common information systems to be effective, it is necessary to define such an ecosystem and determine, which of its elements must be incorporated into an information security plan [70].

#### F10: External environment connections

Information security cannot be viewed as a static concept. Since the external environment is an important impact and dynamic factor, information security must be flexible, responsive, and resilient against the negative impacts of change [56, 88]. External factors of information security comprise social, legislative, and political changes. Specifically, these include market trends, activities of competing organisations, globalization processes, requirements of the international environment, regulatory changes, economic situations, relations in the business or economic sector, security and technological trends [22, 70, 88]. When acquiring new technologies, organisations must act wisely, perform product security tests, andestablish their acceptability [51, 93]. Such analyses are normally conducted in the scope of change management [85], which, according to the professional recommendations [51, 93], require planning and testing of individual changes and their impact on security.

### 2.2 Performing ISec assessment with ISP 10×10M

**Fig 2:**
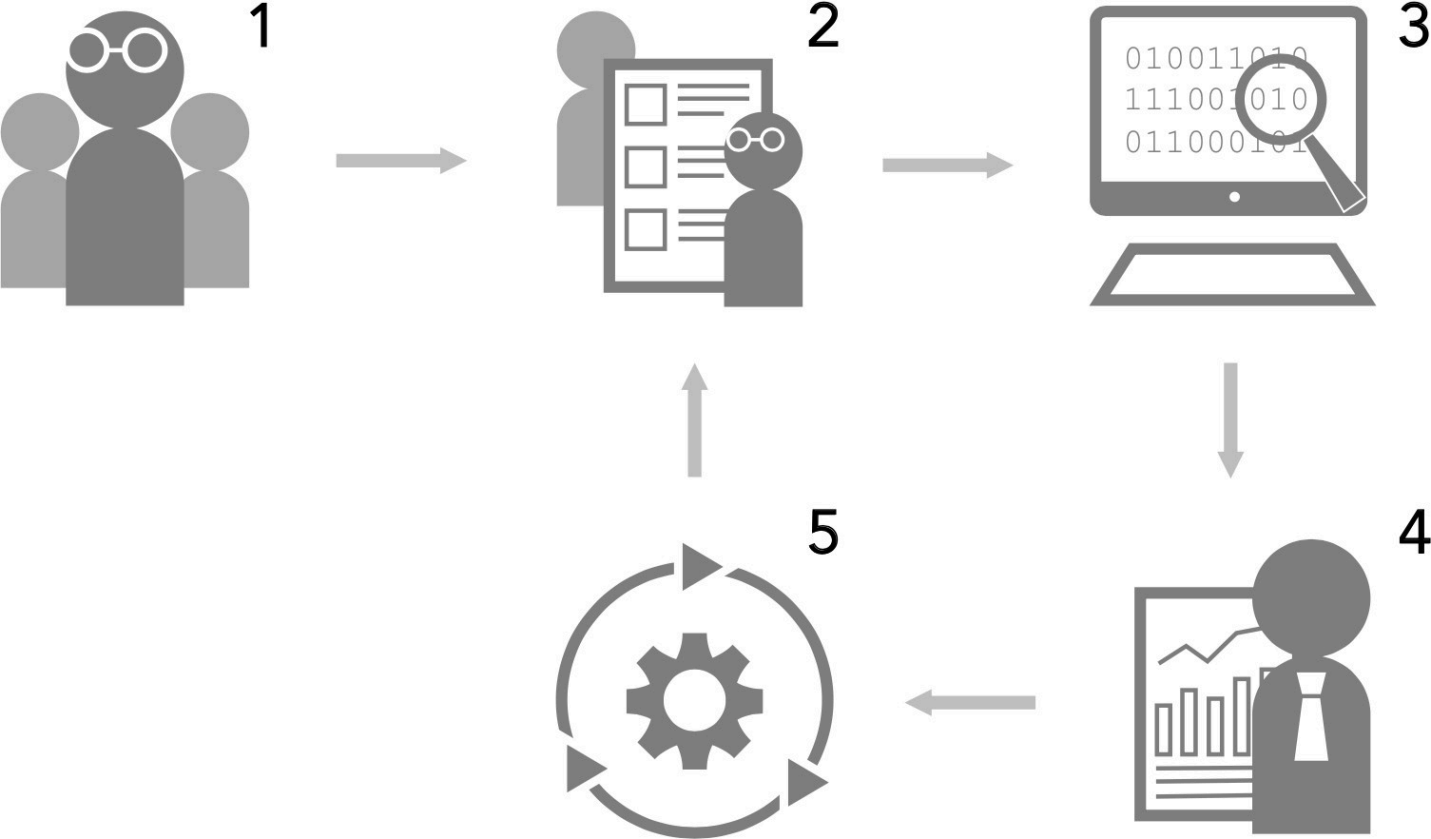
A process suggested for model implementation (1 –delegating the task to ISec experts; 2 –performing an assessment; 3 –data analysis according to the model design; 4 –presentation of the results and identification of gaps in the ISec performance; 5 –implementation of corrective measures).

The model addresses the ISec from a socio-technical perspective. It provides a tool that enables a comprehensive assessment of both social/organisational and technical aspects of ISec. The model should be implemented as follows. The enterprise must first delegate the task of conducting the audit to internal or external ISec experts, establish an evaluation team, and define the scope of the assessment. In the next step, the evaluation team performs an assessment to which degree the measures and activities included in the model (presented in the S1 File) are implemented. There are several methods of acquiring the required data, such as document reviews, observations, and interviews. Since the primary focus of the model is not assessing the technical vulnerabilities and risks (which are commonly identified by vulnerability and penetration tests, event analysis), complex and technical tests are not needed. The data is then analysed according to the model design. The design requires the data to be weighted according to the importance of the particular ISec areas and measures as we proposed in [40]. The obtained results enable to determine the maturity levels of each ISec area as well as the overall performance level of the enterprise. The enterprise therefore can determine the possible gaps in their ISec performance. The process of data and results analysis is followed by the presentation of these results to the top management which decides on the future corrective measures aiming to achieve the desired target level. The correction measures are implemented and monitored for their efficiency. The complete process of ISec evaluation (Fig 2) follows the plan-do-check-act (PDCA) approach which should be a continuous rather than a one-time activity.

## 3 Method

This paperprovides with the validation of the ISP 10×10M model in organisational settings. The research was conducted according to the following procedure:

- sampling of enterprises in the nation-wide database;
- distribution of the participation invitations to the top management of the selected enterprises;
- obtaining consents from participating units and data gathering;
- data analysis including gap analysis and benchmarking;
- presentation of the results to the enterprises.

### 3.1 Measuring instrument

In order to test the model, a questionnaire comprising 105 items was used. The first 100 items are related to ISec measures, while the last 5 items represent the control variables (demographics). The questionnaire consists often ISec areas (factors), while each individual area is further represented by ten items (indicators). Respondents were provided with the following general instruction: “*In the scope of each individual area (from F1 to F10)*, *please assess to which extent your organisation fulfils the criteria or to which extent it would be able to activate the listed measures in the event of a security incident*.” Respondents were asked to evaluate measures on a 5-point Likert-typescale (1 –*Measure has not been adopted;* 5 –*Measure is fully implemented*). The questionnaire also included five demographic items: (1) the level of enterprise’s informatisation; (2) the potential impact of an information incident on enterprise’s reputation; (3) the size of the enterprise; (4) business environment; and (5) the type of business activity. A questionnaire was designed for a self-reported assessment, which is a commonly used lightweight method in ISec scientific research [30, 37–39, 94].

### 3.2 Data collection and sample description

The model is primarily designed to address the needs of smaller or technologically less complex enterprises. The target group, which served as the basis for obtaining the research sample, was defined by the inclusion and exclusion criteria presented in Table 1.

**Table 1:** Inclusion and exclusion criteria for obtaining the research sample.

Inclusion criteria	Exclusion criteria
medium-sized enterprises (50–249 employees)	micro and small enterprises (up to 50 employees)
private sector enterprises	large enterprises (more than 250 employees
	public sector entities
	enterprises with business activities related to providing security, computing and information services
	enterprises undergoing bankruptcy of compulsory settlement procedure
	second and every following enterprise on the list, which was a part of the same business group

An online business directory, i.e. www.bizi.si, which contains all current information regarding legal entities in Slovenia, was used to select the research sample. At the time of conducting the study, the directory contained 970 suitable enterprises that met the inclusion criteria (Table 1). The sample represented 20 percent of the target population. The sampling procedure was based on randomisation which was carried out according to the following procedure. All enterprises were first listed in alphabetical order. Next, every fifth enterprise on the list was selected. The enterprise was excluded if it met the exclusion criteria (Table 1). The excluded entities were replaced by enterprises, which were next on the list. This enabledus to devise a systematic sample, which consisted of a total of 194 units. Online resources were then used to find individual unit’s contact details: first and last names of directors or authorised representatives, postal or e-mail address, and phone number.

Enterprises were then sent an invitation to participate in the study, first in writing and subsequently by e-mail. To boost the response rate, an email reminder to the selected enterprises was sent after the due date expired. The invitation included a clear statement that participation in the study does not require the enterprises to reveal any classified information. Furthermore, this document clarified the concept, objectives, purpose of the research, and stated that participation in the survey was voluntary and anonymous. A special field was dedicated for enterprises to enter a random number of their choice. This number was then used as an identification number to which results were assigned. The results were then privately sent to those enterprises that required the results of their ISec assessment. The document also included a request that the questionnaire should be filled in by the personnel responsible for ISM within the enterprise. The guidelines for the ISec assessment procedure were provided. Consent for participation in the research was obtained by a separate document that was given by the enterprises’ management.

A total of 20 units (10.31 percent response rate) responded to the invitation and submitted an adequately and fully completed questionnaires. The 60 percent of a final sample consisted of enterprises with 50 to 149 employees, while 40 percent of the sample was represented by enterprises with 150 to 250 employees. The 25 percent of all enterprises conducted their business activities exclusively in Slovenia, while the remaining share also operated internationally. Mostenterpriseswere involved in manufacturing activities (40 percent). The following categories included public utilities and energy sector (25 percent), and commercial activities (20 percent). Due to the high degree of diversification, the remaining enterprises (15 percent) were joined under a single category designated “other professional services”. This category includes enterprises that defined their business activity as “road transport and logistics”, “construction and design” or “other professional services”. In the majority of sample enterprises (60 percent) the degree of informatisation was very high while the potential impacts of ISec incidents were reported as even higher since 65 percent of units stated that such incidents could cause a (very) strong negative impact on their business reputation.

## 4 Results

We first analysed the degree to which individual ISec measures are developed. The results were then weighted by using the method we proposed in [40], which is used for evaluating: (1) the performance level of ISec areas, and (2) the overall ISec performance of each individual enterprise.

Before conducting a data analysis, the dataset was checked for missing values. We found that all questionnaires had been fully filled in. First, each individual variable was verified in terms of its normal distribution, which was followed by the assessment of the reliability of each individual factor. The skewness values range between -1.845 and 0.930, while the kurtosis values are between -1.641 and 3.344. The value of only two items exceed 3.0. Due to the small sample size, the aforementioned values enable us to presume that the data are distributed normally, as proposed by [95]. Items were then combined into the corresponding factors (from F1 to F10), which reflect a degree of development of an individual ISec area.

In the next subsections, the results are presented according to ourtwo research questions. The discussion of the paper follows the same structure logic.

### 4.1 Degree of information security areas development

To answer the first research question (RQ1) and determine the degree of ISec areas development, the mean value of every item within an individual area was calculated (the results are presented in the S1 File–Items, means, and medians). These results were then used to calculatethe mean values of ISec areas for observed units. Together with the latter, the overall mean values, as well as the t-tests (calculated using the mean value of the area for the all units combined), Cronbach alpha (CA), and mean inter-item correlation values by the areas (MC) are presented in Table 2. The Cronbach’s alpha (CA) values varied from 0.694 to 0.877, where all factors, apart from one, exceeded the CA value of 0.7, while CA values of four factors were higher than 0.8. The mean inter-item correlation (MC) varied between 0.194 and 0.423, while four factors exceeded the value of 0.3.

**Table 2:** Means, Cronbach’s alpha, mean correlation by factors, and one sample t-test (test value 3, ***—p < 0.001; **—p < 0.01; *—p < 0.05).

Ref.	F1	F2	F3	F4	F5	F6	F7	F8	F9	F10	Overall
**9**	4.1	4.7	4.8	4.6	3.8	4.9	4.8	4.8	4.7	4.2	**4.54**
**719**	5	5	5	4.4	4.4	4.7	3.5	3.5	4.4	3.8	**4.37**
**390**	4.3	4.3	4.4	4.5	3.1	3.8	4	3.4	3.8	3.8	**3.94**
**666**	4.1	4.3	4	4.0	3.1	3.2	4.5	3.6	4.3	3.7	**3.88**
**260**	4.5	3.8	2.6	3.6	3.4	4.6	3.4	4.2	4.4	4.2	**3.87**
**145**	5	4.7	4.7	3.8	2.4	4.5	4.1	1	4.2	3.1	**3.75**
**116**	3.6	4.2	4.3	4.3	3.3	2.9	3.6	3.5	3.6	3.6	**3.69**
**102**	2.7	3.6	3.5	3.2	3.1	4.4	4.2	3.9	3.8	3.8	**3.62**
**916**	4.3	3.4	3.7	3.6	2.5	4	3.5	2.9	4.2	3.7	**3.58**
**246**	3.3	3.5	4.1	3.9	2.8	4.2	3	3.6	3.5	3.7	**3.56**
**988**	4.8	3.7	4	3.7	3.2	3.7	3.5	2	4.2	2.8	**3.56**
**228**	3.4	3.5	3.1	3.9	3	4.2	3.6	3.2	3.6	3.7	**3.52**
**410**	3.7	3.5	3.4	3.1	2.3	3.5	3.9	3.3	3.9	3.5	**3.41**
**400**	3.9	3.7	3.7	3.4	2.1	4.4	3.1	2.3	4.3	2.9	**3.38**
**820**	4.1	3.9	3.8	3.4	2	3.4	4	2.8	3.2	2.6	**3.32**
**983**	3.1	3.7	2.5	3.6	1.9	4.1	2.4	3.3	3.6	3.7	**3.19**
**101**	3.4	3.4	3.2	3.2	2.3	3.3	3.5	3.4	2.8	2.5	**3.1**
**311**	3.6	2.8	2	2.0	1.6	3.8	3.9	3.7	3.3	3.7	**3.04**
**452**	2.4	3.3	3.5	3.3	2.5	2.7	4.4	2.4	3.4	2.4	**3.03**
**214**	3.6	3	3.2	3.7	2	3.8	3.5	1.7	3	2.7	**3.02**
***M***	3.85	3.80	3.68	3.66	2.74	3.91	3.72	3.13	3.81	3.41	
**SD**	0.71	0.57	0.78	0.59	0.70	0.61	0.55	0.89	0.52	0.56	
***t***	5.4***	6.2***	3.9**	5.0***	-1.7	6.7***	5.8***	0.63	7.0***	3.2**	
**CA**	0.83	0.73	0.85	0.74	0.77	0.85	0.72	0.88	0.71	0.69	
**MC**	0.32	0.21	0.34	0.24	0.25	0.39	0.18	0.42	0.21	0.19	

In terms of effectiveness, enterprises reported that F6 (*Organisational culture and top management support*) was the most developed areawith a mean value of 3.91. This area is followed by F1 (*Physical information security controls*) and F9 (*Third-party relationships*). The lowest degree of development was reported for F5 (*Information risk management and incident handling*). Areas F8 and F10, which refer to the *Security management maturity* and *External environment connections*, were also among the less developed areas. The findings of the poorly developed security management (F8) correspond to the observed low degree of management support (related to the items in F6), which can prove to be a hindrance for enterprises. Given the crucial role of management in the process of establishing effective information security, this area (F8) could also act as a potential cause of the poorly developed F5. Furthermore, the relatively low mean values observed in F10 indicate that this is a more complex area, which enterprises tend to develop in the final stages, as it entails activities with long-term effects.

### 4.2 Information security performance

To answer the second research question (RQ1) we analysed the ISec performance of the observed units, and then compared the units with the highest and lowest scores.

First, to establish the overall ISec performance of enterprises, each individual item and area were weighted according to the weights proposed by [40]. The assessment of every individual item was multiplied with the value of the weight specified for that particular item. The values of weighted assessments were subsequently summarized within each individual area, and, finally, the values of all ten ISec areas were summed up. By applying the described method, we obtained the final assessments of individual areas and the overall index for each unit, which corresponds to the result of overall ISec performance.

**Fig 3:**
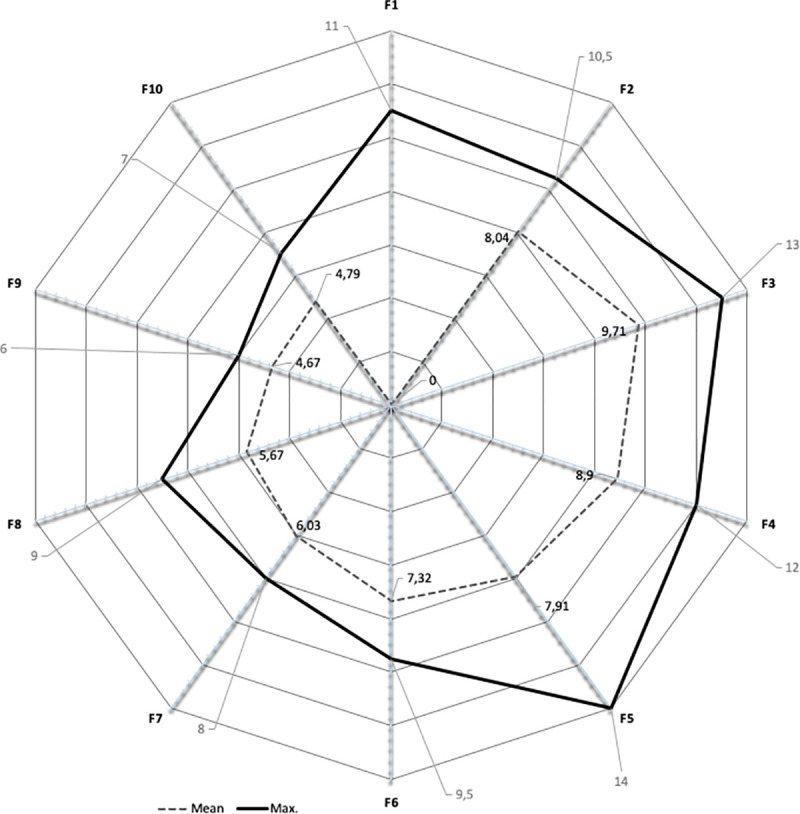
A spider chart representing average absolute values per factor.

The results are presented in Table 3. The columns (*F1– F10*) present the results of units (*Ref*) per individual factors, while the column *Total* shows the overall results. The last column (*Level*) presents the level of ISec performance in the *x*.*y* form, where *x* denotes a principal level and *y* stands for a sublevel. The three rows at the bottom of the table show the mean value of all results (*M*), the maximum score that enterprises could achieve in individual factors (*Max*), and the rate between the mean and maximum values (*Rate*). Furthermore, results for factors (columns *F1-F10*) are presented with two values. Upper value (row *Abs*.) represents an absolute value (score) the unit achieved for a particular factor, while the relative value (row *%*) represents a percentage of this score relative to the maximum value the unit could achieve in the particular factor. A spider chart (Fig 3) provides an additional visualisation of the average absolute values in comparison to the maximum values of the factors.

**Table 3:** Results of information security performance for all units by factors.

Ref		F1	F2	F3	F4	F5	F6	F7	F8	F9	F10	Tot.	Lev.
916	Abs.	9.4	7.2	9.7	8.7	7.1	7.5	5.6	5.2	5.2	5.2	70.9	2.4
	%	85.5	68.6	74.6	72.5	50.7	78.9	70.0	57.8	86.7	74.3		
246	Abs.	7.4	7.3	10.7	9.3	8	7.9	4.9	6.5	4.3	5.3	71.7	2.4
	%	67.3	69.5	82.3	77.5	57.1	83.2	61.3	72.2	71.7	75.7		
214	Abs.	8.4	5.9	8.6	8.9	6.4	7.1	5.9	2.9	3.7	3.9	61.8	2.4
	%	76.4	56.2	66.2	74.2	45.7	74.7	73.8	32.2	61.7	55.7		
400	Abs.	8.4	7.7	9.9	8.5	6.3	8.2	5	4.2	5.2	4.1	67.7	2.4
	%	76.4	73.3	76.2	70.8	45.0	86.3	62.5	46.7	86.7	58.6		
228	Abs.	7.6	7.3	8.1	9.6	8.4	7.9	5.8	5.9	4.4	5	70.0	2.4
	%	69.1	69.5	62.3	80.0	60.0	83.2	72.5	65.6	73.3	71.4		
102	Abs.	5.9	7.7	9.3	7.8	8.8	8.3	6.7	7.1	4.6	5.4	71.6	2.4
	%	53.6	73.3	71.5	65.0	62.9	87.4	83.8	78.9	76.7	77.1		
260	Abs.	10.1	7.9	6.9	9.3	9	8.6	5.5	7.4	5.4	6	76.1	2.4
	%	91.8	75.2	53.1	77.5	64.3	90.5	68.8	82.2	90.0	85.7		
145	Abs.	11	10	12.4	9.1	7.4	8.5	6.7	1.8	5.1	4.4	76.4	2.4
	%	100.0	95.2	95.4	75.8	52.9	89.5	83.8	20.0	85.0	62.9		
410	Abs.	8	7.6	9.3	7.8	6.7	6.5	6.3	5.8	4.7	4.9	67.5	2.4
	%	72.7	72.4	71.5	65.0	47.9	68.4	78.8	64.4	78.3	70.0		
101	Abs.	7.5	7.4	8.4	7.8	6.7	6.2	5.7	6	3.4	3.6	62.5	2.4
	%	68.2	70.5	64.6	65.0	47.9	65.3	71.3	66.7	56.7	51.4		
311	Abs.	8	6.1	5.1	4.7	4.9	7	6.4	6.8	4.1	5.1	58.2	2.3
	%	72.7	58.1	39.2	39.2	35.0	73.7	80.0	75.6	68.3	72.9		
820	Abs.	9	8.2	10	8.2	5.7	6.3	6.4	5.4	3.9	3.7	67.0	2.4
	%	81.8	78.1	76.9	68.3	40.7	66.3	80.0	60.0	65.0	52.9		
116	Abs.	8	9	11.2	10.4	10	5.7	5.6	6.3	4.3	5	75.4	2.4
	%	72.7	85.7	86.2	86.7	71.4	60.0	70.0	70.0	71.7	71.4		
390	Abs.	9.5	9.1	11.6	10.8	8.7	7.1	6.4	6.2	4.6	5.3	79.3	2.4
	%	86.4	86.7	89.2	90.0	62.1	74.7	80.0	68.9	76.7	75.7		
988	Abs.	10.6	7.8	10.4	8.8	9.3	6.9	5.9	3.8	5.1	4	72.7	2.4
	%	96.4	74.3	80.0	73.3	66.4	72.6	73.8	42.2	85.0	57.1		
9	Abs.	8.9	9.8	12.6	11.2	10.8	9.3	7.7	8.8	5.7	5.9	90.7	3.5
	%	80.9	93.3	96.9	93.3	77.1	97.9	96.3	97.8	95.0	84.3		
719	Abs.	11	10.5	13	11	12.2	8.8	5.6	6.1	5.4	5.6	89.3	3.5
	%	100.0	100.0	100.0	91.7	87.1	92.6	70.0	67.8	90.0	80.0		
983	Abs.	7	8.2	6.9	8.6	5.8	7.8	3.9	6	4.7	4.9	63.8	2.4
	%	63.6	78.1	53.1	71.7	41.4	82.1	48.8	66.7	78.3	70.0		
666	Abs.	9	9.2	10.5	9.6	8.6	5.9	7.3	6.7	5.3	5.2	77.4	2.4
	%	81.8	87.6	80.8	80.0	61.4	62.1	91.3	74.4	88.3	74.3		
452	Abs.	5.6	6.7	9.7	7.8	7.3	5	7.1	4.6	4.2	3.5	61.5	2.4
	%	50.9	63.8	74.6	65.0	52.1	52.6	88.8	51.1	70.0	50.0		
***M***		8.5	8	9.7	8.9	7.9	7.3	6	5.7	4.7	4.8		
**Max**		11	10.5	13	12	14	9,5	8	9	6	7		
**Rate** (%)		77	76	75	74	56	77	75	63	78	69		

The results show that enterprises achieved the maximum score only in areas from F1 to F3, albeit only two enterprises got the maximum result in F1 and only one enterprise obtained the maximum score in F2 and F3. Therefore, it is easiest for enterprises to develop physical and technical security, and measures related to information resources management. A similar situation is also observed with respect to the rate between the average and maximum values in individual areas, where, apart from the areas F1 to F3, high rates were also observed for F6 (*Organisational culture and top management support*) and F9 (*Third-party relationships*).

Based on their results, enterprises were categorised into different ISec performance levels. The categorisation involves three principal levels and six sublevels. The categorisation criteria are presented in Table 4.

**Table 4:** Evaluation criteria.

Principal level	1		2		3	
Sublevel	1	2	3	4	5	6
Score	20–30	31–40	41–60	61–80	81–90	91–100

All calculated scores exceed the basic principal level which indicates that all enterprises develop the ISec business function to some degree. Eighteen units were categorised into the intermediate principal level, while only two units were ranked in the first sublevel of the high principal level (3.5).

**Fig 4:**
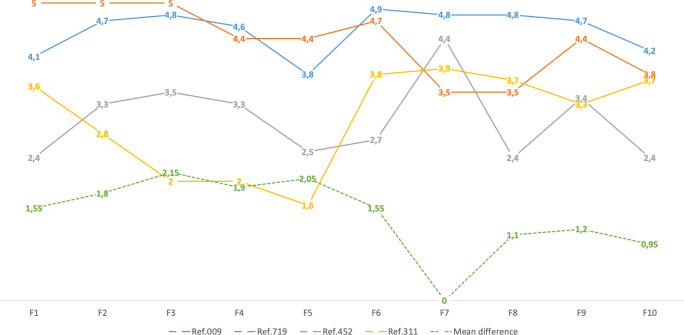
Comparison between the two lowest and the two highest-ranked units by areas with the mean difference.

Second, to identify the key distinction areas, the mean values of the ISec areas (Table 2) of the two lowest ranked units in terms of their performance (Table 3) (Ref. 452 and Ref. 311) were compared with the two highest ranked units (Ref. 009 and Ref. 719). Fig 4 shows the degree of the ISec areas development for all four units and the mean difference between each lowest and highest ranked unit.

The results indicate that the greatest differences in the degree of development occurred in areas corresponding to F2 to F5, while the smaller differences occurred in F7 to F10. Therefore, the greatest differences were observed in the ISec areas corresponding to operational, technical, and information risk-related security measures, while strategic oriented activities seem less diversely developed.

## 5 Discussion

In the following subsection we answer the first research question (RQ1), while the answer to the second research question (RQ2) is presented in the second subsection. The final subsection discusses the theoretical and practical implications of the paper.

### 5.1 Degree of information security areas development

By applying the model, we assessed the ISec situation in organisational settings. We first established the degree of ISec areas development. The interpretation of results obtained in individual areas is provided in a reasonable order, i.e. from the most to the least developed.

We found that the area of *Organisational culture and top management support* (F6) is the most developed one, which is rather encouraging since previous research studies [96–98] on organisational and top management support confirmed its positive impact on the overall level of ISec performance. Herein, we have found that enterprises were well-managing the transparency, cost-efficiency, and optimised the decision-making related to information security. However, they were less successful in terms of involving the top management into various planning procedures, as well as in providing support in the field of human resources and financing. At the same time, research studies presented in the following note that even though most enterprises have already begun to consider information security as a priority, the involvement of top managers in the planning and implementation of activities remains relatively low. Approximately half of organisations provide information on the ISec situation and incidents to the top management [9]. Furthermore, only about one-quarter of organisations include ISec personnel in decision-making bodies, while only one-third of them report that their management have sufficient information at their disposal in order to make a realistic assessment of their organisation’s capabilities and needs [87]. Related to this ISec area, insufficient budgetary or financial support remains a persistent obstacle since it has been reported by various reports for many years [12, 70, 87, 99].

The second most developed area refers to *Physical information security controls* (F1). The enterprises were most effective in controlling the physical environment, as well as in hardware, communication, and power network maintenance. This means that they successfully implemented control over (un)authorised personnel and provided a proper ICT maintenance. On the other hand, enterprises paid less attention to the mobile device management, support systems for critical services and protection against break-ins, intrusions, and wiretapping. Other research studies also find that the lack of control over mobile devices or portable media remains a challenge for information security, which indicates that this is a general issue faced by most organisations [9, 87, 100]. Poorer development of support systems and more complex anti-break-in systems were also to be expected. Given the limited resources and investments, these measures generate relatively high costs which most smaller and financially less stable enterprises simply cannot afford, particularly when considering the above-presented results related to F6. Such measures are usually developed by enterprises that are more security-responsible, complex, and well-resourced.

The third most developed area concerns *Third-party relationships* (F9). This area includes measures, which are mostly related to the management of enterprises’ external links and relationships. Enterprises reported that this area was formally well regulated, which means that they maintain good contractual relationships and well-defined cooperation with their partners and customers, which is a sound basis for inter-organisational security and their overall reputation. The analysis further demonstrated that enterprises very rarely opted for cyber insurance policies and that they rarely conducted security vetting of their partners or involved third parties in the implementation of various procedures (e.g. awareness-raising) and measures in the field of information security. The fact that these measures are less developed is hardly surprising since insurance policies represent only an additional protective mechanism, which helps organisations to recover after ISec incidents. The Global State of Information Security Survey [10, 101] also found that inter-organisational or joint security measures (e.g. verifying information security and related risks in their partner organisations) have been intensively and operationally developed only by approximately half of organisations.

In the area of *Technical and logical security controls* (F2), the area closely related to the F1, the overall result was rather promising. This area took the fourth place on the degree-of-development scale, which means that it belongs to the top half of the most developed areas. The resultsrelated to individual items in this area somewhat complement the conclusions reached in the scope of other ISec areas. We found that enterprises are successful in providing the capacities and capabilities necessary for maintaining information systems. These findings are promising since the appropriate specifications and hardware capabilities fall among the critical conditions for a successful operation of information systems and the smooth, continuous business operations [51, 69]. Apart from system capacity, it is also important that technical and operational measures include protection against malware, which is, according to the results, highly developed. Together with physical protection measures, protection against external ISec threats is one of the very first steps in providing information security [53, 100]. The findings of several studies also show that measures in this area provide the highest return on investment [102]. We also found that enterprises pay a great deal of attention to the regular maintenance and logical protection of their systems. Hence, it seems that enterprises are highly focused on developing the technical aspects of information security. However, despite these generally sound and optimistic results, there is room for improvements. Since we found less promising results in terms of securing stored information and providing secure communications, enterprises should pay more attention to the security of data during their transmission and storage. Ignoring basic measures such as encryption techniques or data/system recovery capabilities increases the chances of information incidents and the abuse of data once they are transmitted from the secure environment.

The area of *Information security policy and compliance* (F7) took fifth place in terms of the degree of development. The results show that items related to fulfilling legal and formal obligations are among the most developed measures, not only in this area but also among all 100 items. We found that compliance with sectoral legislation and honouring the contractual obligations are the most developed measures, while lower results are recorded with respect to meeting international standards and continuous upgrading of security regulations and policies. Other studies report similar findings: in planning their information security, approximately two-thirds of all organisations focus on binding policies [87], while other, namely non-binding policies (for instance, policies dedicated to the management of ISec risks and incidents, recovery policies, etc.) are not being developed in such extent [9].

The overall results show that the areas of *Information resources* (F3) and *Employee management* (F4) took sixth and seventh place. Disciplinary procedures and users’ security vetting seem to be the most underdeveloped measures of the employee management area. Related studies also found that threats originating in the organisation’s internal setting are particularly challenging, since–unlike external threats–they are much more difficult to detect and deal with, which means that despite certain activities aimed at raising awareness among employees, organisations are less successful in managing insider threats [86, 102, 103]. Furthermore, the least developed measures of the information resources management area include activities and controls related to intellectual property protection and security classification of information. Issues related to securing information capital were also observed in the analysis of technical measures (F2). This is important since the failure of enterprises to assess the value and security of information resources can lead to establishing inadequate priorities. In such a case, enterprises can have difficulties in identifying which resources are of key importance and what are their greatest vulnerabilities. The lack or inexistence of capacities to provide adequate protection of confidential data are particularly worrying, albeit comparable to the findings of other studies [10]. The importance of security classification of information, which is a subsequent measure to keeping the information resources inventory, is also highlighted by established international standards (e.g. [52, 53]).

Based on the overall mean values, *External environment connections* (F10) area took eighth place on the development scale. Enterprises report that they manage external changes and pressures successfully, and frequently form strategic alliances with other organisations in their sector. Such connections are advised if enterprises wish to follow trends and manage a dynamic business environment. With respect to information security, such connections enable enterprises to be better prepared for potential legislative amendments or changes in the threat landscape. Less encouraging results were observed in the field of active participation in international settings, which can help enterprises to monitor technological developments and global security trends. Accordingly, it was also observed that enterprises rarely established links with third party advisory and expert groups for the purposes of information-sharing and auditing. Similar findings were reported in the scope of the Global Information Security Survey [70], according to which organisations rarely involve external advisory groups or law enforcement authorities in their response to information security incidents. Furthermore, more than half of the organisations in this survey reported that they did not monitor technological developments and identify potential consequences of changes to their internal security situation. Although our research showed slightly better awareness of enterprises on current development trends, we found inconsistency in following the guidelines and recommendations of specialized third parties. This, when combined with the observed tendency to resolve issues internally, leads to high-risk practices.

The areas of *Security management maturity* (F8) and *Information risk management and incident handling* (F5) took the second last and last place respectively. With respect to F8, we found that enterprises had the most difficulties with the recruiting qualified personnel, which is consistent with the findings related to the analysis of organisational culture and top management support (F6). Results indicate that this is also linked to several other issues, i.e. meetings dedicated to information security seem to be less frequent, while problems also occurred with respect to the long-term and strategic planning, and the lack of formal services or departments that would deal with information security. Issues, such as the lack of professional staff or a dedicated ISec department and continuous communication on ISec issues, must be dealt with immediately and unconditionally. In fact, the lack of systematic organisation and poor management are often reflected in the lack of compliance between operational activities and strategic objectives [59]. Other studies also point to similar organisational challenges, in particular, to poorly defined responsibilities in the field of ISM and the general lack of experts in most organisations [13, 99].

With respect to F5, ISec performance measurement, risk and incident analyses are by far the least developed measures, which is why it is reasonable to presume that enterprises cannot be particularly efficient in the field of ISM. Results related to the use of intelligent threat detection systems and development of crisis management were also less developed, however, in terms of economic analyses, these two measures are among the most profitable and recommended measures [86]. We also found that business continuity policies, incident reporting, and recording procedures were among the slightly better-developed measures. Nevertheless, their mean values remain relatively low compared to the other measures of the model. According to the results we cannot claim that the areas F5 and F8 were properly developed, especially considering that organisations which fail to analyse risks, develop their security management and define relevant roles and responsibilities are the least effective in ISM [59]. Furthermore, the results related to F5 correspond to the findings of other studies, which indicate that merely one-fifth of all enterprises conduct ISec risk reviews, as well as internal and external ISec audits [9]. This, in fact, can lead to a situation where the majority of risks occurring in organisations are overlooked and the response to the incidents is too late or excessively slow [70].

Comparing our results with other researches, it is evident that the final conclusions are similar. In assessing ISM performance several studies confirmed that organisations most often struggle to develop and perform activities related to vulnerability, risks, incidents, procedures, and resources management [30, 31, 33, 39]. The State of Security Operations study [59], found that more than two-thirds of all organisations provide their information security merely through the most basic detection and response capacities. Organisations recorded the lowest results in the areas of organisational structure and processes, while technical measures were among the most developed. The fundamental problem could be sought in the lack of qualified security staff and the low level of staff retention or, in other words, excessive fluctuation, which results in poor security culture and motivation. The Global Information Security Survey [70] conducted among security professionals showed that almost half of organisations require a great deal of work and improvements in the field of ISec development. Moreover, it found that only few organisations were satisfied with the current situation, since more than half only partially met their security needs.

As other studies aimed to determine the common ISec related issues, we have also identified areas and measures which are well- and under-regulated. Naturally, it is inappropriate to generalise the obtained results to all organisations, however, they should be reasonably considered in discussions about progressing the information security in real-world settings.

### 5.2 Information security performance

When drawing the final conclusions regarding the situation in ISec performance, the overall results (i.e. performance scores), which are the product of combining enterprises’ measures evaluation and items’ weights, were used as a basis for classifying units into six maturity levels. Most enterprises (90 percent) were classified into the intermediate principal performance level. One enterprise reached the third sublevel (2.3; Regulated & compliant); while 17 enterprises reached the fourth sublevel (2.4; Formalised & standardised). Only 2 enterprises reached the third principal level (3.5; Evaluated & anticipated). The sixth sublevel was not reached by any enterprise, which means that the research failed to include an enterprise that had developed all measures to this level of maturity. Nevertheless, most enterprises exceed minimum requirements and dedicate certain means and investments to ISM. Moreover, these findings show that enterprises’ focus on ISec efficiency and performance is insufficient. Most enterprises find themselves at the intermediate level, where only the most imperative standards are complied with. The main reason for their stagnation stems from the fact that they are unable to successfully provide organisational support, manage information risks, and keep up with the developing threats and vulnerabilities.

Some of the recent studies also depict a similar picture and report on the relative stagnation of development in this field. For instance, studies show that most organisations (approximately three quarters) seem to attribute significant importance to information security, however, the recommended basic measures are only implemented by approximately half of all organisations [9]. Similar findings are reported in the Global State of Information Security survey [87], which states that most organisations (approximately two thirds) evaluate their ISM capacities as immature, and only fewbelieve that they would be able to detect a more complex cyberattack. Majority organisations also stated that information security fails to meet the organisational needs, while merely four percentbelieved that their strategy is effective and that they adequately controlled threats and vulnerabilities. Furthermore, the Cost of Cybercrime Study [102] showed that ISec measures are implemented at the level of the entire organisation by approximately one-quarter of organisations. The remaining organisations found themselves at the stage of planning, while a somewhat low number of organisations have not implemented any activities. The described situation could also explain why only about a third of all organisations believed that existing investments into information security are effective [99].

According to our findings and the overall scores indicating the ISec performance, the situation in real-world settings is currently under development. There are several shortcomings and room for improvements.

Based on the performed comparison between the two lowest and two highest-rated enterprises, we identified the key distinction ISec areas that determine the strengths of the front-runners. We found the smallest differences in areas related to formal requirements or less cost-intensive measures. As it seems, compliance is the most common driver of ISec development. The widest gap between enterprises was observed in F3, which proves that the ability to identify, assess, and secure key information resources is a prominent aspect that distinguishes front-runners from other enterprises. Therefore, enterprises that do not understand the importance or value of their information capital cannot devise proper ISec plans. A relatively large gap was also observed with respect to F2, F4, and F5, which concern the implementation of technical measures, employee management, and information risk management. These areas are extremely important, as they represent front-line security measures, which prevent ISec incidents. At the same time, they include operationally and financially the most demanding and intense measures.

The ISec areas, in which we found the greatest differences, had also been recognised as the main features of front-runners by previous studies [58, 80, 104]. These studies indicate that the leading organisations in the field of ISM are distinguished from other organisation because they meet the following conditions.

- *The information resources analysis and the ISec performance measurement*. Organisations should be aware of previous incidents, conduct periodic analyses of ISec risks, and be constantly ready to respond to the most likely and critical events. They must have a clear understanding of which parts of information systems are critical and which information resources are of significant importance to their business. They should also monitor security controls with the view of establishing their effectiveness and efficiency. The design and implementation of a proper measuring system to assess the achievement of security objectives are of key importance for the development of information security. This also includes benchmarking in the business sector and establishing the degree of ISec maturity on the basis of established standards. Moreover, organisations must also develop business continuity capabilities, such as response and recovery plans.
- *Technical resources for preventing information security incidents*. Organisations aiming to properly manage ISec threats must prevent both external and internal threats by using appropriate detection capabilities and monitor the compliance with security rules and procedures. In addition, they should also monitor new developments in security measures and invest in those measures that are consistent with their capabilities and needs.

Such a distinguishment of front-runners from other organisations in terms of ISec performance is important particularly in order to identify and advance the most problematic areas which represent challenge for most organisations. A benchmarking enables researchers to emphasise those pivotal areas that should be prioritised both by the organisations themselves and by the key stakeholders responsible for the development of standards and recommendations issued to organisations.

### 5.3 Originality and implications

The originality of this paper lies in addressing several knowledge gaps identified in the related work section of this paper. The current study builds on our previously proposed model and provides its extensive validation. The research was therefore conducted (1) with the modelthat covers the majority of established international ISec standards. The (2) sample of 20 enterprises represents one of the largest samples in the literature, especially when considering that the sampling was done systematically and on a national database of business enterprises. Therefore, enterprises from different business sectors are included in the sample. To the best of or knowledge, it is the first paper that provides an insight into (3) ISec areas that delineate the best performing enterprises from the rest. In the following, we present a more detailed discussion on the contribution, theoretical, and practical implications of this paper.

Firstly, to the best of our knowledge, this is the first attempt to analyse ISec performance from an in-depth socio-technical perspective, including both specific ISec areas as well as ISec measures. Our real-world study confirmed the validity and reliability of the ISP 10×10M model, which is in itself a straight forward and effective tool for establishing the overall situation in terms of ISec performance. We demonstrated a unique quantitative approach and presented the overall degree of ISec areas development, and maturity of individual ISec measures, the significance of which has been recognised by established international standards and previous studies. Such an approach enables the identification of best practices and compliance between activities currently undertaken by organisations and recommended measures. Overall ISec performance scores also allow for the identification of organisations’ actual needs and potentially overlooked ISec areas. Thus, the model is a relatively simple tool to evaluate individual aspects of ISM and the overall performance of organisations’ information security from different perspectives.

Secondly, the identification of differences between the highest and the lowest-ranked enterprises in terms of ISec performance, indicates those areas that pose the greatest challenges in the real-world situation. These areas should thus be given more attention by researchers, particularly in order to identify factors that could contribute to improvements in ISec performance. The findings represent an important point of reference to the key professional stakeholders in developing recommendations and guidelines adapted to the actual industrial needs.

Thirdly, the proposed approach may be applied to a broad range of situations both scientific and practical. From the scientific perspective, the approach proves useful for researchers in the field of IT management and organisational (corporate) security, who perform evaluations of the general state of information security in specific industries or on larger samples of organisations. In practice, research results are primarily useful for professionals responsible for information security within organisations, since they provide an insight into organisational practices and areas that should be dealt with as a matter of priority. Our approach can help managers particularly in the stage of: (a) collecting information regarding short comings in practice, (b) identifying operational needs in the field of ISec, and (c) decision making regarding further steps that would enable adequate development. From the practical perspective, our approach may also be used in case studies, in-house evaluations, or audits. It provides answers to certain questions that cannot be resolved by conducting traditional technical or economic analyses, for instance: how effective is an individual organisation or how does it perform in comparison with other organisations?

### 5.4 Limitations and future work

Like any other, our research is also characterised by certain limitations. Firstly, the principal limitation stems from the size of the sample. Even though the process for selecting the sample was systematic and rigorous, the response rate was only around 10 percent. Secondly, the assessment of ISec performance was conducted in the form of a self-reported study, which is why responses and, consequently, overall ISec performance scores depend on the degree of respondents’ objectivity. Therefore, the reader should be cautious when generalizing the sample results and drawing conclusions.

Since human error can decrease assessment reliability when using self-reported methods, re-survey processes, consistency tests, and a combination of different data are advised [46, 105]. Therefore, future studies using the proposed approach should include multiple respondents, repeated assessments within a single unit (i.e. organisation), and if possible external and independent audits for maximizing the objectivity of the results. Also, due to different business requirements and organisational strategies, extensible models are advised [26]. As a result, a methodology for the adaptation of key performance indicators should be developed to ensure the model’s flexibility, while specific organisational characteristics (such as informatisation level) should be considered in ISec performance evaluation. Moreover, to facilitate its sustainability, the model should be continuously evaluated against its compliance with established ISec standards.

## 6 Conclusion

This paper provides a comprehensive presentation and substantiation of ISec areas that make up the ISP 10×10M model. Furthermore, we presented an approach to ISec performance assessment by addressing technical, organisational, and social aspects. The model was validated in a real-world survey among medium-sized enterprises in Slovenia and provides an overview of the current state of information security. Additionally, and more importantly, it exposes those ISec areas that pose the biggest challenge in the field of ISM.

The observed situation can be described as follows: information security is strategically defined and compliant, however, measures are primarily implemented at technical and operational levels, while its broader and strategic management remains underdeveloped. As it seems, the biggest issues are related to information resources and risk management, where ISec measurement-related activities proved to be particularly problematic. These results thus justify the significance of the aim of this paper.

Even though we found that enterprises do possess certain ISec capabilities and are aware of the importance of information security, their current practices make it difficult for them to keep up with the fast-paced technological and security trends. To achieve the desired level where information security would follow a multidimensional and in-depth approach, we propose the following: (a) strategies and action plans should consider best practices, including the comprehensive protection of the business ecosystem, benchmarking, and ensuring the independence of security management, as well as continuous quality management; (b) there should never be any ambiguity as to who is responsible for information security, its control, and development; (c) there should be no doubt as to the impact of information security on the overall business performance; (d) information security should be defined as an objective in organisations’ vision and business strategy; (e) the continuous monitoring of performance to ensure coordination between security strategies and organisations’ goals; (f) reinforcement of intra-organisational support, both in terms of security intelligence and culture.

With the real-world ISec performance assessment using the proposed approach, it is possible to measure and evaluate the current state of information security in any organisation. Based on such a comprehensive assessment, organisation can perform a rational and systematic decision-making process aiming for the efficient, cost-effective ISec approach and ensure a proactive stance and preparedness for future challenges.

## Supporting information

S1 File Items, means, and medians. (DOCX)
