# Supplementary material for: A real-world information security performance assessment using a multidimensional socio-technical approach
Source: PLoS One. 2020 Sep 8;15(9):e0238739. doi: 10.1371/journal.pone.0238739 (PMC7478844; doi:10.1371/journal.pone.0238739)
Supplement: S1 File — (DOCX) [file pone.0238739.s001.docx]

| **F1: Physical information security controls** | | **Mean** | **SD** | **Med.** |
| --- | --- | --- | --- | --- |
| 1.a | Fire, voltage and flood protection of buildings and premises | 4.05 | .211 | 4.00 |
| 1.b | Adequate installation and management of communication and power network | 4.15 | .209 | 4.00 |
| 1.c | Support systems for critical services – power supply, cooling, communication | 3.40 | .294 | 3.50 |
| 1.d | Control of third-party access to buildings and premises | 4.25 | .239 | 5.00 |
| 1.e | Control of employee access to buildings and premises | 4.40 | .210 | 5.00 |
| 1.f | Adequate installation and physical protection of hardware | 3.90 | .250 | 4.00 |
| 1.g | Regular maintenance of hardware | 4.20 | .172 | 4.00 |
| 1.h | Protection of ICT located outside organizations’ premises (MDM systems) | 3.20 | .296 | 3.00 |
| 1.i | Adequate building architecture and security plan in place – defined security areas | 3.50 | .267 | 3.50 |
| 1.j | Protection of buildings and premises against break-ins and wiretapping | 3.40 | .320 | 3.50 |
| **F2: Technical and logical security controls** | | **Mean** | **SD** | **Med.** |
| 2.a | Malware protection | 4.70 | .128 | 5.00 |
| 2.b | Logical security of programs, systems and databases – identification/authorization | 3.40 | .285 | 3.50 |
| 2.c | Technical protection of local networks (LAN) and network devices | 3.85 | .274 | 4.00 |
| 2.d | Technical measures aimed at protecting information during their storage | 3.05 | .312 | 3.00 |
| 2.e | Technical measures aimed at protecting communications and information during transfer | 3.50 | .276 | 3.50 |
| 2.f | Access control – log management, activity monitoring | 3.55 | .246 | 4.00 |
| 2.g | Adequate system capabilities and capacities for information processing – system reliability | 4.50 | .154 | 5.00 |
| 2.h | Standardization of workstations | 3.80 | .225 | 4.00 |
| 2.i | Change management – analyses of impacts that technology changes have on existing systems | 3.30 | .219 | 3.00 |
| 2.j | Regular (automatic) security updates of software and systems | 4.35 | .182 | 5.00 |
| **F3: Information resources management** | | **Mean** | **SD** | **Med.** |
| 3.a | Security categorization of information | 3.15 | .310 | 3.00 |
| 3.b | Defined administration and other responsibilities related to information management | 3.65 | .327 | 4.00 |
| 3.c | User guidelines for handling information | 3.55 | .294 | 4.00 |
| 3.d | Implementation of the “need-to-know” principle | 3.95 | .185 | 4.00 |
| 3.e | Control over the exercise of administrator and system rights | 4.00 | .241 | 4.00 |
| 3.f | Definition and protection of organization’s intellectual property | 3.10 | .280 | 3.00 |
| 3.g | Definition and protection of personal data | 4.00 | .229 | 4.00 |
| 3.h | Provision of data processing traceability – audit trails | 3.75 | .239 | 4.00 |
| 3.i | Adequate deletion of data, destruction of equipment and physical documentation | 3.30 | .325 | 3.50 |
| 3.j | Information archiving and regular back-ups | 4.30 | .242 | 5.00 |
| **F4: Employee management** | | **Mean** | **SD** | **Med.** |
| 4.a | Raising employees’ awareness regarding information risks and policies | 3.65 | .209 | 4.00 |
| 4.b | Defined user responsibilities related to the use of confidential systems and data | 3.50 | .256 | 3.50 |
| 4.c | Defined disciplinary proceedings, sanctions and infringement proceedings | 3.35 | .264 | 3.50 |
| 4.d | User rights management throughout employment – before, during, after employment | 3.95 | .170 | 4.00 |
| 4.e | Security vetting of employees | 2.50 | .312 | 2.00 |
| 4.f | Employee agreements and declarations concerning the protection of confidentiality | 3.45 | .266 | 3.50 |
| 4.g | Provision of technical and consultative support to employees | 4.20 | .213 | 4.50 |
| 4.h | Defined remote access and teleworking procedures | 4.20 | .258 | 5.00 |
| 4.i | Protection of employee rights during information security control procedures – protection of privacy | 4.15 | .150 | 4.00 |
| 4.j | Professional training of security and technical personnel | 3.65 | .264 | 4.00 |
| **F5: Information risk management and incident handling** | | **Mean** | **SD** | **Med.** |
| 5.a | Business continuity plan and policy | 3.70 | .252 | 4.00 |
| 5.b | Automated early warning systems – IDS, IPS, SIEM | 2.75 | .270 | 3.00 |
| 5.c | Defined procedures for reporting and handling detected irregularities | 3.10 | .289 | 3.00 |
| 5.d | Crisis management – plans for responding to critical security risks | 2.95 | .303 | 3.00 |
| 5.e | An alternative location (i.e. hot spot) for the most important parts of information systems | 2.80 | .304 | 2.50 |
| 5.f | Incident monitoring, recording and analysis – experiential learning | 3.00 | .251 | 3.00 |
| 5.g | Forensic procedures and evidence gathering for incident investigations | 2.30 | .300 | 2.00 |
| 5.h | Information risk management – analysis and evaluation | 2.30 | .263 | 2.00 |
| 5.i | Analyses of former information incidents’ impacts on business operation – damage assessment | 2.35 | .264 | 2.00 |
| 5.j | Assessment of existing security controls’ efficiency – performance measurement | 2.15 | .254 | 2.00 |
| **F6: Organizational culture and top management support** | | **Mean** | **SD** | **Med.** |
| 6.a | Ethical, socially responsible and transparent security management | 4.35 | .209 | 5.00 |
| 6.b | Pursuing the principle of efficiency in information security – economy/cost optimization | 4.10 | .176 | 4.00 |
| 6.c | Good relations and constructive debates regarding security controls between organizational departments | 3.95 | .185 | 4.00 |
| 6.d | Inclusion of information security in the planning of organizational projects and changes | 3.40 | .222 | 3.00 |
| 6.e | Leadership familiarity with security needs – direct communication channels | 4.05 | .235 | 4.00 |
| 6.f | Users’ general satisfaction and confidence with respect to information security | 3.95 | .185 | 4.00 |
| 6.g | Organizations’ innovativeness, excellence and continuous development in the field of information technology | 4.00 | .178 | 4.00 |
| 6.h | Adequate staffing and financial support to information security | 3.50 | .246 | 3.00 |
| 6.i | Clearly defined organizational hierarchy and job classification regarding management of organizational security | 3.90 | .240 | 4.00 |
| 6.j | Leadership involvement in information security planning | 3.85 | .182 | 4.00 |
| **F7: Information security policy and compliance** | | **Mean** | **SD** | **Med.** |
| 7.a | Adoption of a formal information security policy | 3.45 | .312 | 4.00 |
| 7.b | Policy’s breakdown into sub-areas and orderly documentation | 3.30 | .300 | 3.50 |
| 7.c | Monitoring the respect of policies among users during their everyday work | 2.95 | .256 | 3.00 |
| 7.d | Compliance with international standards and recommendations | 3.30 | .272 | 3.50 |
| 7.e | Continuous development and upgrading of information security – control of risks and conformity | 3.30 | .242 | 3.00 |
| 7.f | Regular management reviews and internal audits | 3.80 | .247 | 4.00 |
| 7.g | Compliance with relevant legislation | 4.25 | .160 | 4.00 |
| 7.h | Fulfillment of contractual security obligations | 4.35 | .150 | 4.00 |
| 7.i | Use of licensed products and services | 4.75 | .099 | 5.00 |
| 7.j | Analysis of examples of information security best practices – benchmarking | 3.75 | .204 | 4.00 |
| **F8: Security management maturity** | | **Mean** | **SD** | **Med.** |
| 8.a | Strategic and long-term planning of information security – proactive approach | 2.95 | .266 | 3.00 |
| 8.b | Development of information security as a business function or special department/service within an organization | 3.00 | .363 | 3.00 |
| 8.c | Adequate personnel structure – recruitment of qualified staff | 3.45 | .276 | 4.00 |
| 8.d | Formal authority of security personnel – ability of decision-making | 3.35 | .319 | 4.00 |
| 8.e | Division between system-related and security tasks – separation between IT and security division | 3.10 | .315 | 3.00 |
| 8.f | Cooperation with other organizational authorities in information security planning | 3.30 | .317 | 4.00 |
| 8.g | Regular vertical and horizontal security meetings | 2.55 | .276 | 2.50 |
| 8.h | Team decision-making regarding management of critical security risks | 3.25 | .250 | 3.00 |
| 8.i | Management of employees’ security culture and motivational activities | 3.05 | .235 | 3.00 |
| 8.j | Legitimacy of information security – compliance with user requirements | 3.25 | .239 | 3.00 |
| **F9: Third-party relationships** | | **Mean** | **SD** | **Med.** |
| 9.a | Formalized contractual relationships with partners and suppliers regarding information security | 4.15 | .209 | 4.00 |
| 9.b | Defined security responsibilities with respect to customers | 4.10 | .191 | 4.00 |
| 9.c | Involvement of third parties in the implementation of information security measures | 3.35 | .274 | 3.00 |
| 9.d | Good customer relations – building trust and reputation/organizations’ goodwill | 4.25 | .160 | 4.00 |
| 9.e | Testing ICT before acquisition – defined acceptability criteria and quality | 4.10 | .216 | 4.00 |
| 9.f | Security vetting of business partners and suppliers | 2.90 | .250 | 3.00 |
| 9.g | Defined and regulated security of e-business | 4.15 | .244 | 4.50 |
| 9.h | Adequate technical protection of inter – organizational information systems | 3.90 | .228 | 4.00 |
| 9.i | Formalized contractual relationships for the processing and exchange of personal data | 4.35 | .150 | 4.00 |
| 9.j | Liability insurance covering information security events and incidents | 2.85 | .264 | 3.00 |
| **F10: External environment connections** | | **Mean** | **SD** | **Med.** |
| 10.a | Flexibility of organizations – adapting to changes in the sector and the environment | 3.85 | .221 | 4.00 |
| 10.b | Successful management of competitive and external pressures | 3.65 | .167 | 4.00 |
| 10.c | Cooperation with other sectoral organizations – inter-organizational strategic security ties | 3.65 | .264 | 4.00 |
| 10.d | Participation in economic and business associations, societies and groups | 3.65 | .264 | 4.00 |
| 10.e | Cooperation with competent authorities when dealing with information incidents | 3.45 | .246 | 4.00 |
| 10.f | Cooperation with security consultant groups and external audits of information security | 2.85 | .274 | 3.00 |
| 10.g | Active participation in foreign/international environments – international cooperation for knowledge sharing | 2.80 | .304 | 3.00 |
| 10.h | Defined rules governing communication with the public and competitive organizations | 3.15 | .254 | 3.00 |
| 10.i | Monitoring technological developments and implementing innovations regularly | 3.40 | .234 | 3.00 |
| 10.j | Monitoring and analyzing security trends – development of threats and vulnerabilities | 3.60 | .169 | 4.00 |
